# Impact of Insecticide Resistance on *P. falciparum* Vectors' Biting, Feeding, and Resting Behaviour in Selected Clusters in Teso North and South Subcounties in Busia County, Western Kenya

**DOI:** 10.1155/2020/9423682

**Published:** 2020-04-08

**Authors:** Edward K. Githinji, Lucy W. Irungu, Paul N. Ndegwa, Maxwell G. Machani, Richard O. Amito, Brigid J. Kemei, Paul N. Murima, Geoffrey M. Ombui, Antony K. Wanjoya, Charles M. Mbogo, Evan M. Mathenge

**Affiliations:** ^1^Eastern and Southern Africa Centre for International Parasite Control (ESACIPAC) KEMRI, P.O Box 54840 – 00200, Nairobi, Kenya; ^2^University of Nairobi, P.O Box 30197 – 00200, Nairobi, Kenya; ^3^Machakos University, Machakos Campus, P.O. BOX 136 – 90100, Machakos, Kenya; ^4^Centre for Global Health Research (CGHR) KEMRI, PO Box 1578 – 40100, Kisumu, Nyanza, Kenya; ^5^Vector-borne Disease Control Unit, Ministry of Health, Nairobi, Afya House, Cathedral Road, P.O. Box 30016 – 00100, Nairobi, Kenya; ^6^Jomo Kenyatta University of Agriculture and Training JKUAT Juja, P.O. Box 62 000 – 00200, Nairobi, Kenya; ^7^KEMRI-Wellcome Trust Research Programme, P.O Box 43640 – 00100, 197 Lenana Place, Nairobi, Kenya

## Abstract

**Introduction:**

Behavioural resistance to insecticides restrains the efficacy of vector control tools against mosquito-transmitted diseases. The current study is aimed at determining the impact of insecticide resistance on major malaria vectors' biting, feeding, and resting behaviour in areas with and areas without insecticide resistance in Teso North and Teso South, Busia County, Western Kenya.

**Methods:**

Mosquito larvae were sampled using a dipper, reared into 3-5-day-old female mosquitoes [4944] which were exposed to 0.75% permethrin and 0.05% deltamethrin using World Health Organization tube assay method. Blood meal, species identification, and *kdr* Eastgene PCRs were also performed on adult mosquitoes sampled using mosquito collection methods [3448]. Biting, feeding, resting, and exiting behaviours of field-collected mosquitoes from five selected clusters were analysed.

**Results:**

The lowest *Kdr* genotypic frequency (SS) proportion was found in female *Anophelines* collected in Kengatunyi at 58% while Rwatama had the highest genotypic frequency at 93%, thus susceptible and resistant clusters, respectively. The peak hour for mosquito seeking a human bite was between 0300 and 0400 hrs in the resistant cluster and 0400-0500 hrs in the susceptible cluster. The heterozygous mosquitoes maintained the known 2100-2200 hrs peak hour. There was a higher proportion of homozygous susceptible vectors (86.4%) seeking humans indoor than outdoor bitters (78.3%). Mosquito blood meals of human origin were 60% and 87% in susceptible Kengatunyi and resistant Rwatama cluster, respectively. There was significant difference between homozygous-resistant vectors feeding on human blood compared to homozygous susceptible mosquitoes (*p* ≤ 0.05). The proportion of bovine blood was highest in the susceptible cluster. A higher proportion of homozygous-resistant anophelines were feeding and resting indoors. No heterozygous mosquito was found resting indoor while 4.2% of the mosquitoes were caught while exiting the house through the window. *Discussion*. A shift in resistant *Anopheles gambiae* sl highest peak hour of aggressiveness from 2100-2200 hrs to 0300-0400 hrs is a key change in its biting pattern. Due to the development of resistance, mosquitoes no longer have to compete against the time the human host enters into the formerly lethal chemical and or physical barrier in the form of long-lasting insecticide-treated net. No heterozygous LS mosquito rested indoors possibly due to disadvantages of heterozygosity which could have increased their fitness costs as well as energy costs in the presence of the insecticidal agents in the treated nets. *Conclusions and recommendations*. Out of bed biting by female mosquitoes and partial susceptibility may contribute to residual malaria transmission. Insecticide-resistant vectors have become more endophagic and anthropophillic. Hence, insecticidal nets, zooprophylaxis, and novel repellents are still useful chemical, biological, and physical barriers against human blood questing female mosquitoes. Further studies should be done on genetic changes in mosquitoes and their effects on changing mosquito behaviour.

## 1. Introduction

Endemic areas of stable malaria have altitudes ranging from 0 to 1300 meters around Lake Victoria in Western Kenya and in the coastal regions [1, 2]. Rainfall, temperature, and humidity are the determinants of the perennial transmission of malaria. The vector life cycle is usually short with high survival rate due to the suitable climatic conditions. Transmission is intense throughout the year with annual entomological inoculation rates between 30 and 100 infective bites [3]. The end of June marks the start of the malaria season in east Africa. After the long rains, conditions in lowland swamps and coastal regions are more conducive for mosquito breeding [4–6]. Although, malaria is decreasing through intensified interventions since mid-2000s onwards, these environmental changes might expose population in the highlands of east Africa to an increase risk of malaria and its epidemic particularly if the current interventions are not sustained [7, 8].

Killing adult mosquitoes by spraying living rooms with insecticides have been used successfully in the past [9, 10]. Recently, emphasis has been placed on the use of bed nets that have been treated with a synthetic insecticide to enhance their protection against mosquitoes and the disease they transmit, particularly malaria [11]. Many studies carried out on the efficacy of insecticide-treated bed nets as a means of controlling malaria reported reduced *Plasmodium* transmission rates and clinical malaria after mass ITN campaigns and improved universal coverage [12, 13]. The global incidence and mortality rate of malaria have fallen by 37 and 58%, respectively, in the last decade [14]. Unfortunately, the remarkable ability of insect population to develop resistance to most of the classes of insecticides often leaves control programs with few insecticide options [7, 15]. Thus, the monitoring and management of insecticide resistance are important in malaria control programs. The early detection of resistance is a vital part of resistance management because it may lead to the development of insecticide use strategies that would minimize the rate of evolution resistance [16, 17].

To block or diminish the harmful impact of insecticidal compounds, the female mosquito can adopt a wide range of behaviours [18]. The first line of defence is qualitative behavioural resistance whereby mosquitoes avoid temporal, spatial, or topical contact with insecticides. In regions with massive use of indoor residual spraying (IRS) or ITNs, selection may favour foraging later in the morning or earlier in the evening, periods when the human hosts are not protected by indoor treatments or bed nets [19]. As insecticidal compounds become more widely used, it is probable that anophelines may not be able to avoid coming into contact. In this case, if mosquitoes are not immediately killed after insecticide exposure either due to sublethal doses or physiologically resistant genotypes, then they could theoretically evolve quantitative behavioural resistance [20]. In different insect species, behavioural quantitative resistance may include curative self-medication, resistance-promoting behavioural thermoregulation, and or escape reactions that reduce contact duration with the insecticide. These behavioural activities could limit the insecticide's direct negative effects following nonlethal exposures the same way biochemical metabolic resistance lowers the amount of insecticidal active ingredients reaching target sites. Female mosquitoes can adaptively evolve in response to selection pressures imposed by insecticide tool use as long as sufficient genetic variation in constitutive behavioural resistance traits and/or inducible behavioural resistance traits accumulate in a given population [18, 21]. Induced or plastic resistance traits occur within a generation whereas constitutive resistance traits occur when genetic variants spread through the population over generations. Better matching phenotypes as a result of phenotypic plasticity can allow organisms to produce different phenotypes in response to different environmental conditions [22, 23]. Experimental and theoretical studies propose that when constant conditions such as high overall risk of contact with an insecticide and widespread ITN coverage, constitutive behavioural resistance is expected whereas when environmental conditions are variable such as when the risk of contacting the insecticide is unpredictable perhaps because of heterogeneous ITN coverage, the evolution of phenotypic plasticity or inducible behavioural resistance will be favoured [22]. Nevertheless, induced and constitutive resistance traits are not mutually exclusive, hence can coexist in a given population. Therefore, at the individual level, some behavioural resistance traits for example, early biting can be fixed and others plastic, as in the case of zoophagic mosquitoes. Some individuals may display constitutive resistance traits while others display plastic resistance traits at population level [18, 24–27].

Increased outdoor host-seeking or zoophagy is also consistent with mosquito behavioural adaptation to the use of ITNs and IRS. Infective malaria vector-human host contact moments during an infective bite by a mosquito which thereafter finds a safe cool resting place to successfully digest the blood meal thus allowing *P. falciparum* to fully develop in its gut. Once the blood meal is digested, further infective blood feeding is occasioned, hence heightened malaria transmission in a given region while the mosquito finally exit safely to the oviposition and breeding sites [28, 29]. The biting behaviour of anophelines is an important determinant of malaria transmission. Understanding the local vector host-seeking behaviour, its outdoor/indoor biting preference, and nocturnal biting periods is essential for effectively applying and improving vector control methods, such as long-lasting insecticidal nets (LLINs) and personal protective measures. The spread of malaria and any efforts to control it may be influenced by a small number of people who are bitten and infected frequently [30, 31]. The interference with preference and frequency by a female mosquito to bite noninfected persons and or animals over infected persons may be a game changer as far as decline in active malaria transmission cases is concerned.

Finally, physiological and behavioural resistance may likely coexist in natural mosquito populations, and it will be important to study the possible trade-offs and associations between these different protective strategies. Quantification and characterization of the contributions made by insecticide resistance towards successful infective bites, longevity, and oviposition or lack of it in the light of bed net use and indoor spraying as the current vector control interventions may add more impetus towards informed responsive quality insecticide resistance management and monitoring as Kenya enters malaria preelimination phase [1]. The present study sought to investigate the impact of phenotypic and genotypic resistance levels on major malaria vectors' biting patterns and feeding and resting behaviour in areas with and areas without insecticide resistance in Teso North and Teso South, Busia County, Western Kenya.

## 2. Materials and Methods

### 2.1. Study Site

Busia County is found in Western Kenya and it borders Uganda to the west, Bungoma County to the north, Kakamega County to the east, and Lake Victoria and Siaya County to the south. Main economic activity is trade with neighbouring Uganda through Busia town. Away from towns, the country side heavily depends on fishing and agriculture in maize, cassava, sweet potatoes, beans, and millet and sugarcane being the main cash crops. Busia County is made up of seven subcounties, namely, Matayos, Funyula, Nambale, Butula, Teso North, Teso South, and Bunyala. Teso North and Teso South had 20 locations or clusters of which we randomly selected five clusters: two clusters, namely, Rwatama and Kengatunyi in Teso North, and three clusters, namely, Kaliwa, Odioi, and Akiriamasit in Teso South. Purposive sampling of the Teso subcounties was due to the recent reports from Busolwe and Tororo districts in eastern Uganda near the border with Kenya where a high frequency of the *kdr* allele (1014S) was documented (Mawejje et al., 2013). The high *kdr* allele frequency in Uganda was similar to what had been observed in the Asembo study site in Kenya, which is approximately 150-200 km to the southeast of Kenya.

### 2.2. Sample Size Determination

Sample sizes were dependent on WHO assay requirements whereby susceptibility tests were performed on non-blood-fed females, aged no more than 3-5 days postemergence. One hundred and fifty adult females were used, 100 of which were exposed to the insecticide (in 4 replicates each of around 25 mosquitoes). The remaining 50 served as “controls” (i.e., 2 replicates, each of around 25 mosquitoes). For positive control and negative control, respectfully, 50 susceptible KISUMU Asembo strains of Anopheles mosquitoes were exposed in WHO tubes with insecticidal papers and 50 females exposed in WHO tubes with untreated papers. A sampling frame of household list from county registration files gave us the total number of households in each randomly selected sublocation or cluster, village, and compound, respectively. A clustered probability sample was achieved with the help of computer generated tables of random numbers which were used to select sublocation, village, compounds, and households where mosquito sampling was carried out. Selected houses lay within 2 km radius from larval collection sites. The sample size obtained was 96 houses, hence twenty houses per cluster or sublocation. Larval collection sites were randomly selected within 2-3-kilometre radius from selected households.

### 2.3. Mosquito Collection Methods

Both larvae and adult stages of mosquito were collected for two years after year one baseline susceptibility survey. Larvae were collected from their natural breeding sites using standard dippers, put in plastic containers, and transported to the laboratory for rearing, species identification, and susceptibility tests. Only *Anopheles* larvae were retained in the containers as screening was done on all collected larvae using morphological features described by Gilles and Cortzee 1987. Adult mosquitoes were collected using indoor resting vacuum aspiration, human landing catch, window exit traps, pyrethrum spray catch method, and outdoor pot collection. Anopheline mosquitoes were identified morphologically as *An. gambiae sensu lato (s.l.)*, *An. funestus*, other *Anopheles*, and non-*Anopheles* and characterized by gonotrophic stata (empty, blood-fed, gravid, and half gravid female mosquitoes). Blood-fed abdomens were preserved in a freezer maintained at -18°C and stored for use in blood meal PCR. All legs and wings were preserved in Drierite and stored for use in *kdr* gene and species identification. Adult sampling was done at the end of the long (May-July) and short rains (Oct-Nov) from 2012 to 2014.

#### 2.3.1. Indoor Resting Vacuum Aspiration

A vacuum aspirator was used, either a motor-driven one or a manually operated using suction pressure in the mouth. Collected adult mosquitoes were selected and put in paper cups and transported to the laboratory in a cooler box. Sucrose solution in moderately soaked cotton wool was placed on the paper cup net as food to the adult mosquitoes.

#### 2.3.2. Window Exit Trap (WET)

To collect mosquitoes that bite indoors but rest outdoors in order to determine the effect of resistance on the normal movement and feeding habits of mosquitoes, window exit traps were used. In each study cluster, a total of five houses were randomly selected for window traps; an index house was randomly selected and additional houses were selected based on proximity to the index house. The window traps were placed over the windows of bedrooms at 6.00 pm. In the morning, the trapped mosquitoes were collected from the traps using a mouth aspirator. Window exit traps were most suitable for fitting only to rooms that are well sealed and that had few exit points for mosquitoes. Other openings other than the window to which the trap was fitted were covered or blocked with dark clothes or cartons except the eves. Usually, the sleeping room was selected and the trap well fitted to a window. Parts of the window not covered by the trap were covered with dark cloth, cartons, or hardboard. The trap was fitted in such a manner that the collecting sleeve pointed outward. It was important to fix traps into position well before sun set. Mosquito collection was done the following morning just after sunrise. All anopheles mosquitoes were collected through the sleeve of the trap with the use of a mouth aspirator. Separate paper cups were used to transport the live and dead mosquitoes collected from each house. Household data was also entered in a structured questionnaire. The paper cups were clearly labelled in pencil or pen with at least the following essential information: location, date, exit trap number, house number or householder code, time of collection, whether mosquitoes were found dead or live in the trap, and name of the collector.

#### 2.3.3. Pyrethrum Spray Catch (PSC)

To obtain the indoor resting densities of mosquitoes, pyrethrum spray collection method was done in all the houses that had window traps the previous night. Collection took place between 6 am and 8 am. Inhabitants in the house were asked to wait outside, during the procedure. The number of children and adults who reportedly slept in the house the previous night was recorded, and presence of ITNs/LLINs was recorded. All food items and drinking vessels were removed from the house. White sheets were spread on the floor and over the furniture within the house. Two collectors, one inside the house and one outside, sprayed around the eaves with 0.025% pyrethrum emulsifiable concentrate with 0.1% piperonyl butoxide in kerosene. The collector inside the house then sprayed the roof and walls. The collector outside the house sprayed through the eaves ahead of the collector spraying inside the house. The house was closed for 10 minutes after which dead mosquitoes were collected from the sheets and transferred to the laboratory on moist filter paper inside petri dishes.

#### 2.3.4. Human Landing Catch (HLC)

Female mosquitoes were attracted to humans as they quested for blood meals. The number of mosquitoes biting or landing on humans is a major determinant of malaria transmission. The suitable locations for the night collections were selected in such a way that they were closer to the vector breeding sites in the area. Direct collection of biting mosquitoes was performed during the night when malaria vectors were active for they take blood meal in the night. In a full night programme, hourly collections were made during the entire period from 17.00 hrs to 07.00 hours, therefore, from dusk to dawn. Being a very laborious activity, two teams of collectors were used, each team working half of the night, both indoor and outdoor. In the case of outdoor collections, rainy hours when it was not possible to collect were also recorded. HLC was done for two consecutive nights per month and the method involved a consented adult sitting down with legs exposed and waiting for mosquitoes to come feed on the collector where he or she used a mouth aspirator to collect the blood questing females and placed them in netted paper cups with a central upper hole blocked with cotton wool. Alupe Sub-District Hospital in the Teso South and Moding Health Centre in the Teso North subcounties provided medical supervision for all the collectors as for the other members of the community.

#### 2.3.5. Outdoor Pot Collection (OPC)

Clay pots are often used for storing drinking water in the homes in the study area. The clay pots were locally designed, made, and placed outdoor from 18:00 to 06:00 hrs at about 5 m from the house (Odiere et al., 2007 and Degefa et al., 2019). Each pot was about 20-litre capacity, with an opening of 20 cm width, a round bottom, and a maximum width of 45 cm. A 2 cm diameter hole was placed into the center of the base during manufacture. The hole made the pot useless to hold water, hence limiting likelihood of theft. Mosquitoes were collected from the pots once in the morning from 06:00 to 09:00 hrs by placing a cloth mesh from a standard adult mosquito cage on the opening and secured as described by Odiere et al., 2012. One of the two samplers then lifted the pot to expose the opening to light and agitate mosquitoes inside and blew into the small hole at the bottom, causing the mosquitoes inside the pot to take flight and enter the cage being held into position by the second sampler. The cloth mesh was then removed, and remaining mosquitoes in the pot were recovered with an aspirator and transferred to the cage, completing the collection.

### 2.4. DNA Extraction and Species Identification

DNA was extracted from the legs and wings of *An. gambiae sl* and *An. funestus* complexes using ethanol precipitation method (Collins et al., 1987). Conventional polymerase chain reaction (PCR) was used to distinguish between the two sibling species of the *An. gambiae s.l.* species complex native to Western Kenya, *An. gambiae* s.s. and *An. arabiensis* (Scott et al., 1993).

### 2.5. WHO Susceptibility Assays

Mosquito larvae samples were reared and exposed to World Health Organization (WHO) susceptibility kits impregnated with 0.75% permethrin, 0.05% deltamethrin, and 0.1% bendiocarb insecticides. The WHO protocol was used for testing susceptibility to permethrin, deltamethrin, and bendiocarb insecticides. Treated test papers with the WHO diagnostic dosages were supplied by the WHO Collaborating Centre in Kenya. Cohorts of 25 female mosquitoes were exposed to different insecticides at temperatures of 25 ± 2°C and 70–80% relative humidity following the standard WHO tube test protocol [14]. Negative and positive controls were exposed to untreated and treated filter papers for 1 hr, respectively. Knockdown time was recorded after every 10 minutes during the 60-minute exposure. After 1 hr exposure, mosquitoes were transferred to recovery tubes and maintained on 6% sucrose solution for 24 hrs. Mortality was recorded after 24 hr recovery period. Mosquitoes that were knocked down after 1 hr exposure and those that were alive after the 1 hr exposure and still surviving 24 hrs later were collected and stored individually in 95% alcohol for subsequent molecular analysis.

### 2.6. Genotyping for Kdr Mutations

DNA was extracted from adult *An. gambiae* and *An. arabiensis* mosquitoes as earlier described (Scott et al., 1993). Real-time PCR was used to quantify the genotype at amino acid position 1014 of the voltage-gated sodium channel, following the methods of Bass et al., 2007 as modified by [24]. Samples were genotyped for the wild-type (susceptible) allele using probe 5′-CTTACGACTAAATTTC-3′ and for the 1014S *kdr* allele using probe 5′-ACGACTGAATTTC-3′. Real-time- (RT-) PCR reactions were done using Strategene MxPro 3000 machine using a 96-well format.

### 2.7. Detection of Blood Meal Sources Using Polymerase Chain Reaction (PCR)

The abdominal section of blood-fed *Anopheles* mosquitoes was cut transversely between the thorax and the abdomen. Genomic DNA was extracted from mosquito abdomens using ethanol precipitation method as described by Collins et al. One universal reverse primer and five animal-specific forward primers (human, cow, goat, pig, and dog) were used for amplification of cytochrome b gene, encoded in the mitochondrial genome to test for specific host blood meal origins using conventional PCR.

### 2.8. Data Collection, Management, and Analysis

WHO criteria for susceptibility are as follows: mortality rates between 98% and 100% indicate full susceptibility; mortality rates between 90% and 97% require further investigation while in mortality rates <90%, the population is considered resistant to the tested insecticides. Means in the experimental clusters were compared with the means in positive and negative controls as well as between clusters. All analysis was performed in SAS version 9.4 (SAS Institute, Cary, NC). The frequency of the resistance genotype and allele was calculated using the Hardy-Weinberg equilibrium test for *kdr* genotypes and *kdr* alleles. Exit rates were calculated as the number of mosquitoes captured in the window exit traps divided by all mosquitoes collected (window trap, indoor resting aspiration, and PSC) expressed per cluster. A contrast between susceptible and resistant clusters was made in terms of human vector biting, blood index, blood meal preference, and resting behaviour. Vector biting behaviour was referred to the number of mosquitoes collected during the human landing catches analysed by hour of collection to determine the time of night that most biting occurred, summarized by clusters in the subcounties. Human blood index was the proportion of blood-fed mosquitoes that fed on humans calculated as the number of samples positive for human blood divided by the total number of mosquitoes tested.

More information on materials and methods used in this study is found in my linked paper [32] published @ “https://www.hindawi.com/journals/jpr/2020/3560310/”;

## 3. Results

### 3.1. Phenotypic and Genotypic Resistance Levels for all Clusters and Teso North and Teso South Subcounties

Female mosquitoes sampled in Odioi cluster were the most phenotypically resistant to permethrin while Kengatunyi vectors were the most phenotypically susceptible (Table 1). Rwatama had the highest proportion of resistant SS genotypic frequency while female anophelines collected from Kengatunyi had the lowest resistant SS genotypic frequency. Levels of resistance in susceptible Kengatunyi cluster were statistically different from levels of resistance in resistant Rwatama cluster (*p* < 0.05). Genotypically, homozygous SS genotype carrying mosquitoes were resistant to insecticides while the homozygous LL carrying genotypes were susceptible. More results on species composition and insecticide resistance are explored in Githinji et al., 2019 [32].

### 3.2. Biting Patterns for all Clusters and Teso North and Teso South Subcounties

**Figure 1:**
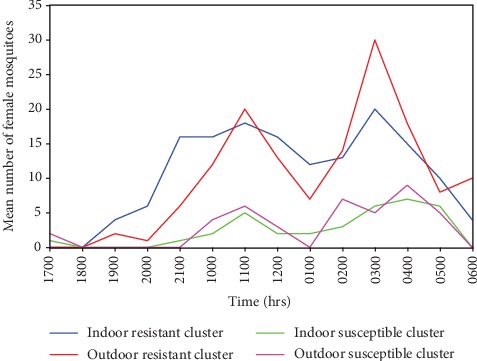
Biting patterns in female mosquitoes collected indoor and outdoor in susceptible and resistant clusters in Teso North and Teso South subcounties in Western Kenya.

Major peak biting hours were 0300-0400 hrs in resistant cluster and 0400-0500 hrs in susceptible one (Figure 1). There was a higher number of mosquitoes questing for blood in the resistant cluster than in the susceptible cluster. Outdoor biters were more than indoor biters throughout the night. Mosquitoes were more active in the mornings than in the evening.

**Figure 2:**
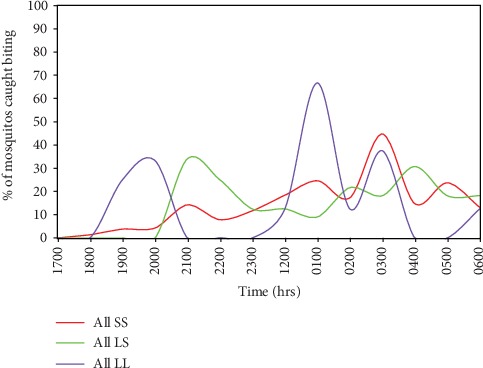
Biting patterns in homozygous-resistant, heterozygous-resistant, and homozygous susceptible female mosquitoes collected in Teso North and Teso South subcounties in Western Kenya.

Homozygous SS genotype carrying vectors had an unimodal 1700 hrs to 0700 hrs biting pattern; heterozygous LS mosquitoes had a bimodal biting pattern while recessive LL *Anopheles gambiae sl* had a multimodal biting pattern with sharp fluctuations from hour to hour (Figure 2). Susceptible vectors were earlier biters than the heterozygous LS and homozygous SS biters. Heterozygous LS female mosquitoes maintained the all-time 2200 hrs peak biting hour. All SS, LS, and LL carriers were biting at dawn.

During dusk and dawn, there were lower proportions of homozygous-resistant genotype carrying female *Anopheles* mosquitoes (83.4%) as compared to 87.5% indoor human biters (Table 2). There were also more resistant exophagic *Anopheles* vectors (82.8%) at bed time than during nonbedtimes (66.7%). It was very interesting to note that the proportion of homozygous susceptible and heterozygous-resistant vectors seeking for a human blood meal was the same indoors (13.3%) during out of bed times of the day and outdoor (8.6%) during bed time hours of the night. There was a higher proportion of outdoor female mosquito seeking human blood meal during bed time hours (82.8%) than during dusk dawn times (66.7%). The vectors were more endophagic than exophagic during both 1700-2200/0500-0700 hrs (83.4%) and 2200-0500 hrs (87.5%).

### 3.3. Abdominal Statuses

The clusters with the highest proportions of fed, unfed, and gravid are Akiriamasit, Odioi, and Rwatama, respectively (Table 3). The susceptible cluster Kengatunyi had a higher proportion of unfed while the resistant cluster Rwatama had the higher proportion of fed and gravid female mosquitoes. Exiting mosquitoes were mostly unfed. There was a significant difference between the proportions of fed mosquitoes collected through pyrethrum spray catch as compared with fed vectors sampled through WET and OPC (*p* < 0.05).

### 3.4. Source of Blood Meals

**Figure 3:**
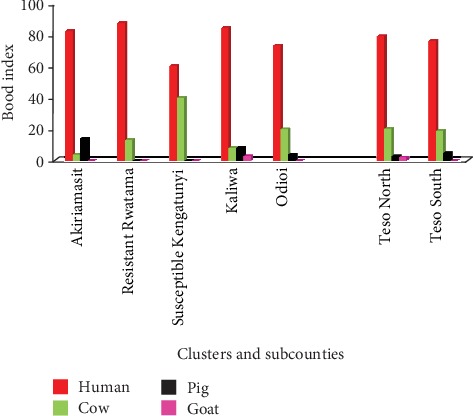
Host blood meal preferences in female Anopheles mosquitoes sampled from different clusters in Teso North and Teso South subcounties, Western Kenya.

Human blood meal remained the most preferred (Figure 3). The highest human blood-fed vectors were found in the cluster with the highest insecticide resistance while the lowest proportion of human blood-fed mosquitoes were caught in the cluster with the lowest insecticide resistance. None of the analysed blood meals had been sourced by the mosquitoes from dog, cat, donkey, and chicken. Vectors from low resistance cluster had the highest affinity for bovine blood. Pig's blood was also a preference among female mosquitoes collected in another low resistance cluster by the name Akiriamasit.

The proportion of mosquito blood meals that were of human origin was the highest in the female mosquitoes collected from the resistant cluster at 87% while the lowest proportion was found in the vectors sampled from the susceptible cluster at 60% (Table 4). Eighty per cent of female anophelines carrying resistance conferring SS genotype were human blood positive while 50% of susceptible LL genotypic malaria vectors fed on human blood. All Kengatunyi anophelines exited through the window of the hut or house. All homozygous susceptible (LL) malaria vectors exited; they were also the least human blood seekers. Heterozygous (LS) anophelines had a higher human blood index and a lower exit rate than homozygous (SS) vectors.

### 3.5. Resting Behaviour

**Figure 4:**
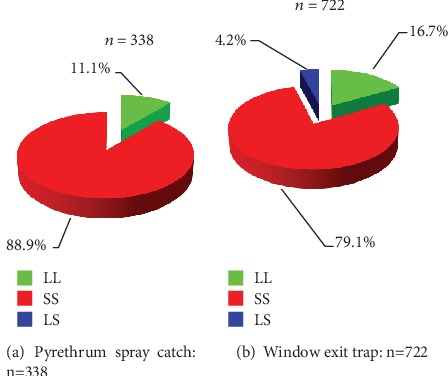
Impact of insecticide resistance on feeding and resting behaviour in female *Anopheles* mosquitoes collected in Teso North and Teso South subcounties in Western Kenya. (a) Pyrethrum spray catch on endophagic and endophillic behaviour. (b) Window exit trap on endophagic and exophillic behaviour.

Proportion of resistant female *Anopheles* mosquitoes which were resting inside the houses was the highest while homozygous susceptible genotype carrying mosquitoes made up 11.1% (Figure 4(a)). There were no heterozygous-resistant female mosquitoes feeding and resting indoors. Homozygous-resistant malaria vectors feeding indoors and exiting through the window were the highest in proportion followed by the homozygous susceptible mosquitoes while the least were heterozygous-resistant ones (Figure 4(b)).

## 4. Discussion

The interplay between several physiological resistance mechanisms in response to insecticidal agents' usage including biochemical (e.g., target site modifications and metabolic), morphological (e.g., cuticular thickness), and behavioural resistance mechanisms is also emerging as an important topic of research which may lead to discoveries aiding the design of resistance managements strategies. A shift in resistant *Anopheles gambiae* sl highest peak hour of aggressiveness from 2100-2200 hrs to 300-400 hrs past midnight is a key change in its biting pattern. Due to the development of resistance, mosquitoes may no longer have to compete against the time the human host enters into the formerly lethal chemical and or physical barrier. Instead, the vector keeps away from the hours the human host is awake and hostile to its bites to the hours when the human host is immobilized and most vulnerable to bites due to sleep. Biting through the net have been reported in the major malaria vector, *Anopheles gambiae* [33]. Towards dawn when the human host is awake and active again, the biting rate went down but not as low as during early hours of night. Burning different kinds of vegetation or wood as fire wood while preparing dinner in the evening or breakfast in the morning has been known to act as a repellent in Wosera District of Papua New Guinea [34]. Outdoor biting exceeded indoor biting mosquitoes possibly as a result of repellent effect of permethrin treated nets and also behavioural resistance promptings [35]. This indeed confirms that insecticidal nets are still effective as physical, chemical, and repellent barriers to malaria vectors. Residual malaria transmission may be taking place more at dawn than at dusk, depending on numbers of infective bites. Cross border early in the morning activities as goods and services are smuggled across the border may have lead the vector to change from its former biting patterns.

The number of attempted bites in highly resistant cluster exceeded those of lower susceptibility cluster. Susceptible mosquitoes were more of early biters than late biters while resistant females were more of late biters than early biters. Smoke has been known to repel mosquitoes questing for a human blood meal, hence a deep in biting volumes during cooking hours of the night [36]. More hungry-resistant mosquitoes were active in seeking a blood meal as early as 1700 hrs while unfed susceptible mosquitoes could be seen attempting a successful bite as late as 700 hrs in the morning. [29] reported that not only were infected mosquitoes more likely to take a second blood meal than their uninfected counterparts, they were also disproportionately drawn to infected hosts hence heightened malaria infective bites.

The current study found out that resistant vectors had higher sporozoite rate than susceptible ones, and unfed females were higher in proportion among susceptible than among mutants. Resistant strains of mosquitoes preferred human blood, hence fed and rested indoors. Different biting rates of mosquito are required by both the malaria parasite and its mosquito to ensure parasite transmission success and mosquito reproductive success, respectively. Attempts by both partners, that is the vector and the *Plasmodium*, to maximize their successes constrain the trade-off existing between mosquito biting rate and survival. Effect of human and mosquito circadian rhythms on each other may lead to constitutive or induced physiological processes and activities to maximize their survival rates [19, 27]. Changes witnessed in the biting, feeding, and resting patterns may have led to enhanced transmission and defense system, hence abundant survival of both host and parasite [19, 30, 37].

Infection by malaria parasites does not harm their mosquito vectors. On one hand, fecundity of the mosquito increases with increased rate of blood feeding because of the strong positive correlation that exists between the amounts of blood imbibed by the mosquito and the number of eggs it lays [19, 27, 38]. On the other hand, blood feeding increases the risk of mosquito mortality since mosquitoes are likely to be killed by the irritated vertebrate host while feeding or trying to feed or by predators due to mosquito's increased body mass and decreased speed in flight. Therefore, mosquito reproductive success and *Plasmodium* transmission success is a function of mosquito biting rate [39]. Therefore, parasite's success is maximal at a biting rate that is higher than required for maximal mosquito's success, as long as transmission increases more rapidly with fecundity and biting rate.

The LL curve showed three high peak hours at 1800-2100 hrs, 2400-200 hrs, and 300-400 hrs. Acute fluctuations could have been a result of constitutive or induced behavioural adaptation due to constant or varied knock down effects, physical–chemical barrier and, repellency action by insecticidal nets. LS curve was smoother as an intermediary but the smoothest curve was shown by SS carrying female mosquitoes whose more definite biting rhythms seem undeterred and undisturbed throughout the night, an indication of resistant malaria vectors' tolerance to physical barrier, insecticidal, and repellence effects.

Resistant *Anopheles gambiae* ss are still highly anthropophagic. Opportunistic *An. arabiensis* was almost as zoophagic as anthropophagic. Resistant vectors were more endophagic and endophillic than exophagic and exophillic while LS and LL female mosquitoes were more exophagic and exophillic than endophagic and endophillic. No LS vector rested outside possibly due to disadvantages of heterozygosity which could have increased their fitness cost as well as energy cost in the presence of the insecticidal agents in permethrin treated nets [39]. SS mosquitoes were biting more during bed time while LS and LL vectors were seeking a human blood meal more during out of bed hours of the dusk to dawn period than during bed time. Temperature has been known to majorly alter mosquito and parasite life-history traits, a temperature sensitive combination which determine malaria transmission intensity [40, 41]. Temperatures, humidity, and wind do vary from hour to hour throughout the night. To inform control and prevention strategies and estimate malaria risk, outdoor and not indoor mean temperature should often be considered.

But, host preference is normally affected by a myriad of extrinsic and intrinsic factors. Inherent factors are determined by genetic selection, which appears to be controlled by adaptive advantages that result from feeding on certain host species. Host preference of mosquitoes, although having a genetic basis, is characterized by high plasticity mediated by the density and accessibility of host species, which by their abundance form a readily accessible source of blood. Host selection behaviour in mosquitoes is an exception rather than the rule. Those species that express strong and inherent host selection behaviour belong to the most important vectors of infectious diseases, which suggest that this behavioural trait may have evolved in parallel with parasite-host evolution [18, 20, 42, 43]. Energy costs during down regulation of insecticidal active ingredients through enzyme activities may have increased the aggressiveness in female mosquitoes for a host blood meal long before dusk [44, 45]. Fitness costs may substantially shorten lifespan, prevents insemination and egg production, and significantly blocks *Plasmodium falciparum* development, three components that are crucial to malaria transmission [34].

## 5. Conclusions and Recommendations

Most resistant malaria vectors fed and rested indoors while susceptible ones fed and rested outdoors, an important development during active residual transmission. Insecticidal nets are still useful repellent, chemical and physical barriers against human blood questing female mosquitoes, hence may be up scaled even further. Bed nets and indoor residual spraying may not completely cut off infected malaria vector-human contacts. Larval source management, sterile mosquito techniques, and novel mosquito repellent topical applications may protect humans from out of net mosquito bites at dusk and dawn. Further studies should be done on genetic manipulation and phylogenetic trends in malaria vectors and their effects on changing biting, feeding, and resting patterns. Effects of resistance genes on overall mosquito behaviour may be genetically and molecularly analysed, characterized, and quantified. Valuable genetic determinants of these traits should be identified through genomic analysis searching for signatures of selection in the genomes of mosquito vectors and their sibling species sequenced in the last few years. Weather driven models of probabilistic changes in malaria transmission intensity may now be boosted with mosquito changing behaviour-driven models to estimate malaria risk and also predict epidemics of mosquito borne diseases.

## Figures and Tables

**Table 1:** Phenotypic and genotypic resistance levels in *Anopheles gambiae* sensu latu collected from five clusters from Teso North and Teso South subcounties of Busia County, Western Kenya between 2012 and 2014.

Clusters	Phenotypic resistance levels (% mortality)			Genotypic resistance levels (%)		
	Permethrin	Deltamethrin	*n*	SS	LS	LL
Kaliwa	75 ± 5.4	78 ± 15.6	223	91.5	2.7	5.8
Odioi	57 ± 19.4	66 ± 19.1	424	76.9	4.7	18.4
Akiriamasit	71 ± 15.4	75 ± 11.8	216	76.9	13.9	9.2
Kengatunyi	86 ± 5.7	86 ± 18.3	85	57.6	7.1	35.3
Rwatama	66 ± 23.1	78 ± 8.0	148	93.9	3.4	2.7
Mean	71	77	291.2	79.4	6.9	13.7
Teso North	67.6	73	449	78.6	9.4	12.0
Teso South	76	82	647	82.0	4.0	14.0

WHO criteria for phenotypic susceptibility are as follows: mortality rates between 98% and 100% indicate full susceptibility; mortality rates between 90% and 97% require further investigation while in mortality rates < 90%, the population is considered resistant to the tested insecticides.

**Table 2:** Impact of insecticide resistance on bed and nonbedtime hours' human biting patterns in indoor and outdoor environments for female mosquitoes sampled from Teso North and Teso South subcounties in Western Kenya Human Landing Catch (HLC) (*n* = 605).

Time (hrs)	Indoor or outdoor	LL	SS	LS
1700-2200/0500-0700 hrs (7 hrs of nonbedtime)	Indoor (%)	13.3	83.4	13.3
	Outdoor (%)	12.5	66.7	20.8

2200-0500 hrs (7 hrs of bed time)	Indoor (%)	5.4	87.5	7.1
	Indoor (%)	8.6	82.8	8.6

**Table 3:** Percentages of abdominal statuses of female mosquitoes as per cluster, method of collection, and subcounties sampled from Teso North and Teso South subcounties in Western Kenya between 2012 and 2014.

Clusters	Fed	Unfed	Gravid
Kaliwa	6.2	49.4	44.4
Odioi	8.2	74.3	17.6
Akiriamasit	19.0	48.0	33.0
Kengatunyi	6.6	63.2	23
Rwatama	9.2	31.5	59.2
Mean	9.84 ± 5.2619	53.28 ± 16.26	35.44 ± 16.7615
Methods of collection			
PSC	32.5	24.4	43.2
WET	1.0	62.9	36.1
OPC	0	53.7	46.3
Subcounties			
Teso North	1.3	39.7	58.8
Teso South	11.2	60.5	28.3

**Table 4:** Human blood indices and exit rates in female mosquitoes sampled in five clusters from Teso North and Teso South subcounties in Busia County, Western Kenya.

Clusters	*n*	Human blood index	Exit rates (%)
Kaliwa	59	83	77
Odioi	251	72	69
Akiriamasit	207	81	37
Susceptible Kengatunyi	104	60	100
Resistant Rwatama	324	87	81
Total	1016	76.6 ± 10.7842	72.8 ± 23.0261
Genotype			
LL	58	50	100
LS	283	100	93
SS	15	80	96
Total	356		

## Data Availability

The datasets supporting the conclusions of this article are available upon request.

## Abbreviations

AR: *Anopheles arabiensis*
DNA: Deoxyribonucleic acid
ELISA: Enzyme-linked immunosorbent assay
GA: *Anopheles gambiae*
HLC: Human landing catch
IRS: Indoor residual spraying
ITN: Insecticide-treated net
*Kdr*: Knock-down resistance gene
LL: Homozygous recessive or susceptible
LLIN: Long-lasting insecticidal net
LS: Heterozygous resistant
PCR: Polymerase chain reaction
PMI: President's malaria initiative
PSC: Pyrethrum spray catch
SS: Homozygous resistant
UN: Universal primer
WET: Window exit trap
WHO: World Health Organization.
